# Magnetic Properties and Spontaneous Polarization of La-, Mn- and N-Doped Tetragonal BiFeO_3_: A First-Principles Study

**DOI:** 10.3390/ma11060985

**Published:** 2018-06-11

**Authors:** Qiuhong Tan, Qianjin Wang, Yingkai Liu

**Affiliations:** 1 College of Physics and Electronic Information, Yunnan Normal University, Kunming 650500, China; tanqiuhong1@126.com (Q.T.); liuyingkai99@163.com (Y.L.); 2 Yunnan Provincial Key Laboratory for Photoelectric Information Technology, Yunnan Normal University, Kunming 650500, China

**Keywords:** multiferroic, BiFeO_3_(BFO), first-principles, magnetic properties

## Abstract

Multiferroic materials have been receiving attention for their potential applications in multifunctional devices. Chemical substitution is an effective method for improving the physical properties of BiFeO_3_ (BFO). However, different experimental results have been reported for Lanthanum- (La-) and Manganese (Mn) -doped BFO ceramics. Here, we systematically studied the magnetic properties and spontaneous polarization of La-, Mn-, and Nitrogen (N) -doped tetragonal BiFeO_3_ using density functional theory with the generalized gradient approximation and U-value method. The calculated results demonstrated that the systems show ferromagnetism with Mn and N doping, whereas no magnetization was found with La doping in G- and C-type antiferromagnetic orderings. Our research further revealed that the ferromagnetism is attributed to the *p*-*d* orbital hybridization. Berry-phase polarization calculations predicted a large polarization of 149.2 µC/cm^2^ along the [001] direction of pure tetragonal BFO. We found that La and N substitution had little influence on the spontaneous polarization, whereas Mn substitution reduced the spontaneous polarization. The reduced energy barrier heights of the doped systems indicate the reduced stability of the off-centering ferroelectricity against the thermal agitation. These findings provide greater understanding for controlling and tuning the multiferroic properties of BFO.

## 1. Introduction

Multiferroic materials are receiving considerable attention for their potential applications in multifunctional devices [1,2]. BiFeO_3_ (BFO) is the most promising candidate for practical applications among ABO_3_-type perovskites due to its multiferroic properties at room temperature (T_C_~1100 K and T_N_~640 K). However, some serious shortcomings of BFO limit its practical applications in devices, such as high leakage current and weak net magnetization. Therefore, numerous studies have been undertaken to solve these problems. Chemical substitution is an effective method to improve the performance of BFO and many experimental studies have been reported on the A- and B-site substitution of BFO [3,4,5,6,7,8,9,10,11,12,13,14,15,16,17,18,19,20,21,22,23,24]. Rare earth and transition metals are employed to enhance the multiferroic properties of BFO with their substitution at the A- and B-site, respectively. Among them, lanthanum (La) and manganese (Mn) elements doped at the A-site and B-site of BFO, respectively, have been extensively investigated. However, different experimental results were reported in La- and Mn-doped BFO thin films. Some experimental investigations reported that La-doped BFO films enhanced ferroelectric remnant polarization and improved ferromagnetic properties [8,17,18,19]. However, You et al. [20] reported that La doping destabilized the in-plane polarization component, and Lazenka et al. [21] reported that La-doped BFO film showed weak ferromagnetism. Besides, some groups [22,23] reported that the Mn-doped BFO system showed intrinsic weak magnetism, whereas other groups [24,25] observed enhanced magnetism in Mn-doped BFO thin films. Therefore, a systematic theoretical investigation was necessary to clarify these issues.

High-strain BFO thin film has a tetragonal structure, which was reported in many experimental and theoretical studies [26,27,28,29,30]. The tetragonal structure is not only suitable for improving the application of device miniaturization but may also enhance the multiferroic properties. Therefore, in this work, the multiferroic properties of La and Mn substitution at the A-site and B-site of tetragonal BFO were investigated by using first-principles. Additionally, the multiferroic properties of nitrogen (N) substitution at the O-site of tetragonal BFO was also investigated as a new method of improving the multiferroic properties of BFO because room temperature ferromagnetism was reported in N-doped BaTiO_3_ and SrTiO_3_ ferroelectric materials [31,32]. Our work highlights the explicit physical mechanism of the multiferroic properties of La-, Mn-, and N-doped BFO films and provides useful guidance for improving the multiferroic properties of BFO.

## 2. Computational Details

**Figure 1:**
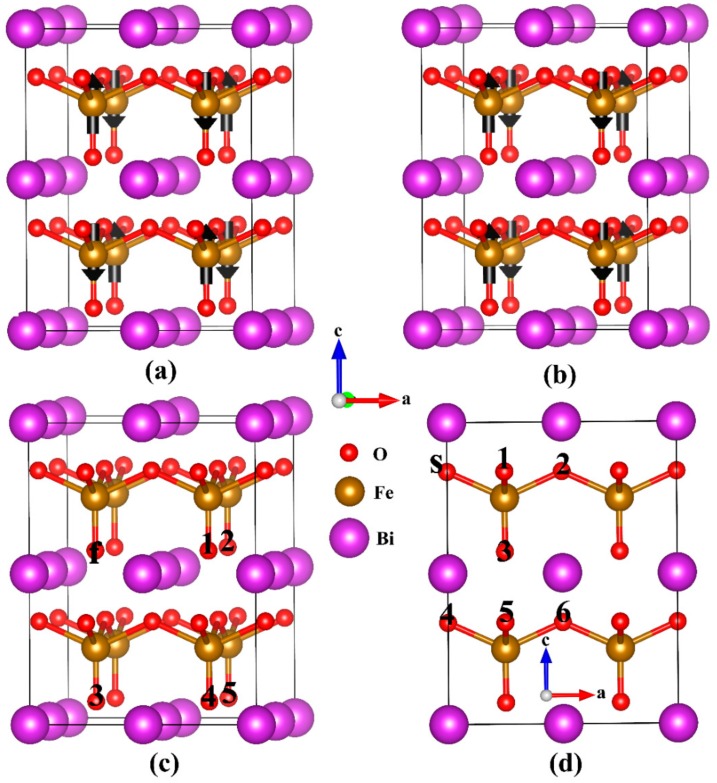
The 2×2×2 tetragonal BiFeO_3_ (BFO) supercell used in our simulations. (**a**) G-antiferromagnetic (AFM) and (**b**) C-AFM spin arrangements. (**c**,**d**) The non-equivalent configurations as two nitrogen (N) atoms were placed on two oxygen (O) sites in the supercell. The arrows on the iron (Fe) ions indicate the spin arrangement. O atoms that will be substituted by N atoms are denoted by numbers.

The structural optimization and electronic structure calculations were carried out by using the plane-wave pseudopotential techniques developed in the QUANTUM-ESPRESSO code [33]. We used Pardew-Burke-Ernzherof (PBE) [34] parametrization for the ultra soft generalized gradient approximation (GGA) pseudopotentials and PBE+U functional. The PBE functional usually provides reasonable lattice parameters compared to experiments, even if it is less accurate for structural optimization than some specialized GGAs [35,36,37,38]. As such, we explicitly treated 11 valence electrons for La (5*s*^2^5*p*^6^5*d*^1^6*s*^1.5^6*p*^0.5^), 15 for Mn (3*s*^2^3*p*^6^3*d*^5^4*s*^2^), 5 for N (2*s*^2^2*p*^3^), 15 for Bi (5*d*^10^6*s*^2^6*p*^3^), 8 for Fe (3*d*^7^4*s*^1^), and 6 for O (2*s*^2^2*p*^4^) atoms. A Hubbard-*U* scheme with a typical value of *U* = 4.5 eV [39,40] was used on the La, Mn and Fe atoms for the better treatment of the localized transition metal *d* electrons. In the calculation, we used a kinetic energy cutoff of 30 *R*y; Monkhorst-Pack *k*-point grids [41,42] of 3×3×3 were used for structure optimizations and then the denser 4×4×3 *k*-points were adopted for the total energy calculations. The Methfessel and Paxton Fermi smearing method with a smearing width of 0.01 *R*y was used [43]. The calculation conditions were checked to obtain converged results. The ions were relaxed until the interatomic forces on them were less than 10^−3^*R*y/bohr. Both *G*-type and *C*-type antiferromagnetic (AFM) spin orderings in tetragonal BFO were investigated in our calculation since many first-principles and experimental works reported that the two magnetic structures nearly degenerate [29,44,45,46]. The optimized lattice constants of tetragonal BFO were *a* = 3.77 Å and *c*/*a* = 1.30 Å, which agreed well with the experimental results [30]. A 2 × 2 × 2 supercell containing eight BFO formula cells for tetragonal structure was built based on the optimized lattice constants, as seen in Figure 1a,b.

## 3. Results and Discussion

We first investigated the magnetism of La, Mn, and N substitution at the A-, B-, and O-site of BFO, respectively. The calculated magnetic moments were both one μ_B_ for Mn- and N-doped in BFO with G-AFM and C-AFM orderings. However, La dopant did not affect magnetization because no electron was added or removed for La^3+^ replacing Bi^3+^. Thus, the reported apparent improvement inmagnetism in La-doped BFO is not caused by La substitution but originates from some other intrinsic defects [47]. N dopant caused the redistribution of charge among Fe, O, and N ions. However, N is not an ideal trivalent in our calculation, causing the N ion and neighboring O ions to have magnetic moments (0.12 μ_B_ for one N and 0.45 μ_B_ for all O ions) and the antiparallel two Fe ions to have nonequilibrium magnetic moments (3.96 μ_B_ and −3.53 μ_B_, respectively). To investigate the magnetic coupling between magnetic ions, two Fe (or O) ions were substituted by Mn (N) atoms in the supercell. In our earlier work [48], we investigated the magnetic coupling between two Mn ions in tetragonal BFO. Results indicated that Mn atoms prefer to assemble and exhibit a parallel spin alignment with a total moment of 8 μ_B_. The robust ferromagnetism was attributed to the stronger *p*-*d* hybridization between Mn-3d, Mn-4p, and neighboring O-2p orbitals. For N substitution at the O-site, there are two nonequivalent O ions (i.e., the apical and basal plane atoms of the inverted pyramid structure) in BFO; thus, we discussed two cases when two N replace two O ions in the G-AFM and C-AFM magnetic lattices. The first case contained five nonequivalent possible positions (i.e., C_f1_–C_f5_ configurations) and the second case contained seven nonequivalent possible positions (i.e., C_S1_–C_S6_ and C_34_ configurations), as seen in Figure 1c,d. The calculated results are listed in Table 1 and Table 2. From the table, we can see that the magnetic properties of N-doped BFO are related to the distribution of N atoms. We found that the C_S3_ configuration is favorable in terms of energy and shows robust ferromagnetic coupling with a total magnetic moment of 2 μ_B_.

**Figure 2:**
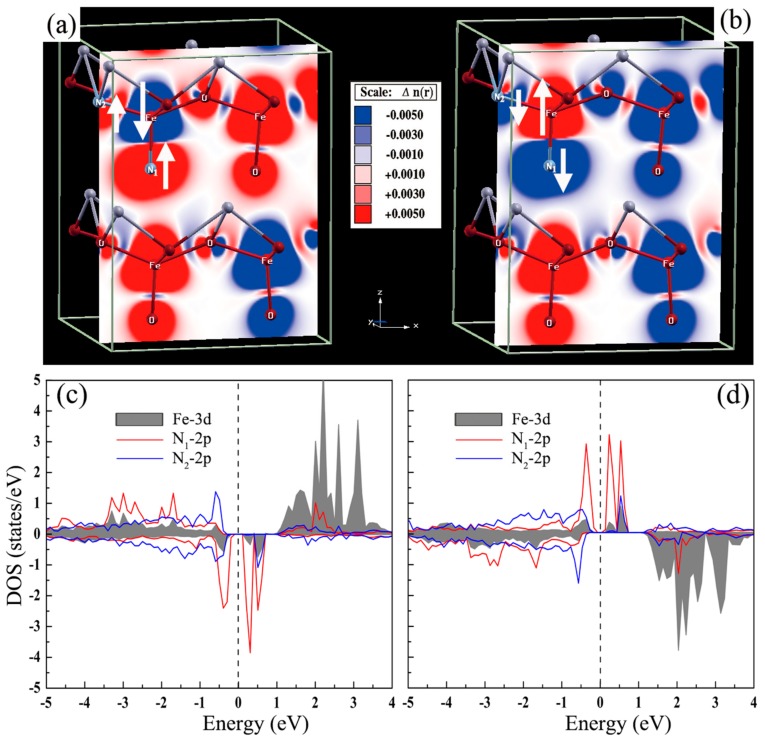
Spin-density distribution of (**a**) G-AFM and (**b**) C-AFM orderings in N-doped tetragonal BFO supercell. (**c**,**d**) The partial DOS of Fe and N ions corresponding to (**a**,**b**), respectively. The red and blue regions denote the spin-up and spin-down charge densities, respectively.

We also investigated the origin of ferromagnetism in terms of electronic structure. Figure 2a,b display the spin charge density of the C_S3_ configuration in G-AFM and C-AFM orderings, respectively. The parallel spin alignment of the two N ions is mediated by the neighboring Fe ion, which is in antiparallel spin alignment with the N ions. Figure 2c,d show the partial density of states (DOS) of Fe and N ions corresponding to Figure 2a,b, respectively. As can be seen in Figure 2c,d, there is an overlap between Fe 3*d* states and N 2*p* states at the Fermi level, which indicates the stronger interaction between Fe 3*d* electrons and N 2*p* electrons. This further reveals that the ferromagnetic coupling originates from the *p*-*d* hybridization. The C_S1_ and C_S2_ configurations in which the two N ions substitution at the equivalent positions and link to the same Fe ion own a net magnetic moment of 0 μ_B_. Further investigation found that the two N ions are not spin polarized and thus show nonmagnetic. The N-N pairing interaction leads to a nonmagnetic state that is attributed to the *ppσ* bonding state and *ppπ* states, which accommodate all 10 electrons and caused the *ppσ* antibonding state to be empty [49].

**Figure 3:**
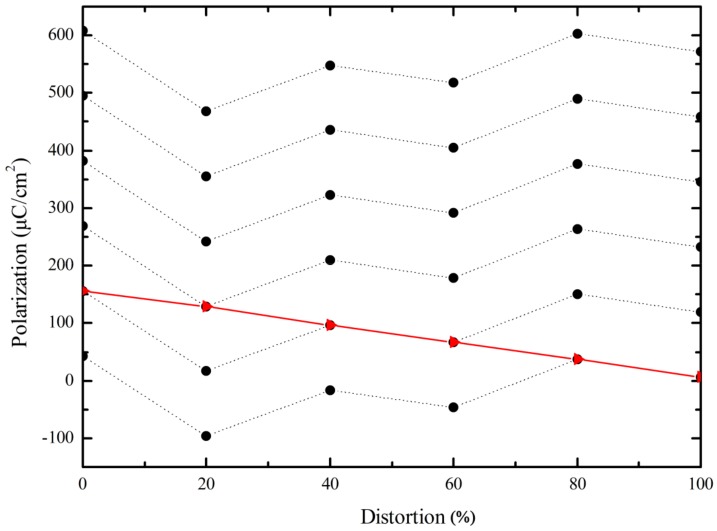
Calculated polarization as a function of percentage of distortion from the centrosymmetric structure (0% distortion) to +*P*4*mm* structure for BFO. The black dots are the calculated polarization at small steps of *λ* for the different crystals considering the polarization quantum jump, and the red line is the real evolution of polarization with distortion.

**Figure 4:**
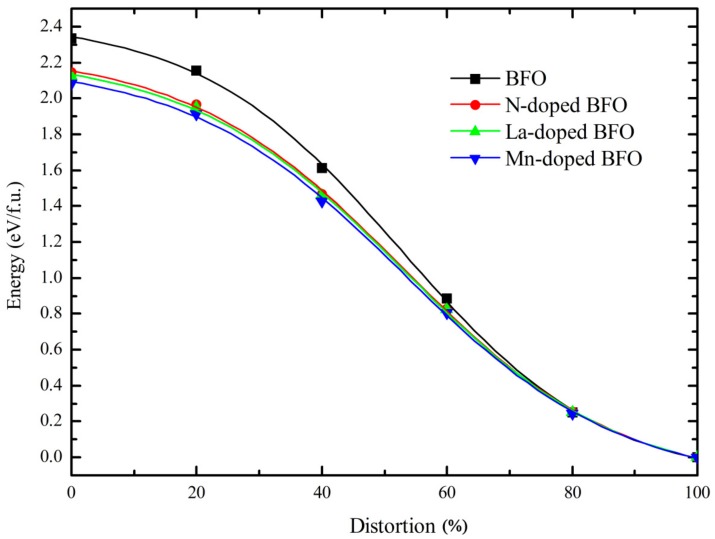
The total energy as a function of percentage of distortion from the centrosymmetric structure to +*P*4*mm* structure for pure and doping tetragonal BFO.

We then calculated the spontaneous polarization of La, Mn, and N-doped tetragonal BFO respectively based on the Berry-phase theory of polarization [50,51]:

$$P(\lambda )=\underset{i}{\sum }\frac{eZ^{i}r_{i}(\lambda )}{\Omega }-ie\underset{v}{\sum }\int_{BZ}\frac{dk}{{\left(2\pi \right)}^{3}}\langle u_{v}^{\lambda }|\nabla u_{v}^{\lambda }\rangle$$

where $Z^{i}$ is the atomic number of the *i*th atom, $u_{v}^{\lambda }$ are the Bloch wave functions, Ω is the volume of the unit cell, and the integral is performed over the first Brillouin zone. For comparison, the spontaneous polarization of pure tetragonal BFO was also calculated. In order to obtain the spontaneous polarization of the ferroelectric phase of tetragonal BFO, the non-polar reference state (centrosymmetric structure, *λ* = 0, labeled with 0% distortion) and ferroelectric state (*λ* = 1, labeled with 100% distortion) were selected as the end points. We inserted several intermediate structures along an idealized “switching path” between the non-polar reference state and the ferroelectric state. Considering the polarization quantum *eR*/Ω, which was 113 µC/cm^2^ along the [001] direction in BFO unit cell, to jump between branches of the polarization lattice, *e* is the electronic charge, *R* is the lattice vector in the direction of polarization, and Ω is the volume of the unit cell [50,51]. The spontaneous polarization is the difference between the two values of end points on the same branch of polarization lattice. Figure 3 shows the calculated polarization value for pure tetragonal BFO. The red line denotes one real evolution of polarization with distortion, and the spontaneous polarization was calculated by 156.1−6.9 = 149.2 µC/cm^2^, which agrees well with other theoretical calculated results [26] and experimental results [52,53]. Therefore, this method can be credibly used for spontaneous polarization predictions of La-, Mn-, and N-doped BFO with G-AFM and C-AFM orderings. Figure 4 shows the calculated total energy as a function of percentage distortion from the centrosymmetric structure to the ferroelectric state (+*P*4*mm* structure) for BFO. Energy varied continuously from the maximum (centrosymmetric structure) to the minimum (+*P*4*mm* structure). Therefore, we speculate that the energy curve will be a symmetrical as the distortion from centrosymmetric structure to the −*P*4*mm* structure with opposite polarization will form a double-well potential. The barrier height of the double-well potential was about 2.335 eV, which is in good agreement with the experimental results reported in BFO films grown on La-doped SrTiO_3_ substrates [53].

Table 3 shows the calculated results for one La-, Mn-, and N-doped BFO at the A-, B- and O-site, which correspond to doping concentrations of 12.5%, 12.5% and 4.17%, respectively. From Table 3, the values for spontaneous polarization for La- and N-doped BFO almost did not changes in C-AFM ordering and only changed minimally in G-AFM ordering compared with pure BFO. For Mn dopant, the value of spontaneous polarization tended to decrease, especially in C-AFM ordering, where the value reduced to 123.8 µC/cm^2^. Notably, the barrier heights of the double-well potential all reduced for La, Mn and N doping, which reduced the stability of ferroelectricity against thermal agitation. Thus, the reported apparent improvement in ferroelectric polarization by La doping is not caused by the enhanced off-centering ferroelectric polarization but originates from the *P*4*mm* tetragonal structure, which is transformed from rhombohedral structure with increasing La doping concentration [54,55,56,57].

**Figure 5:**
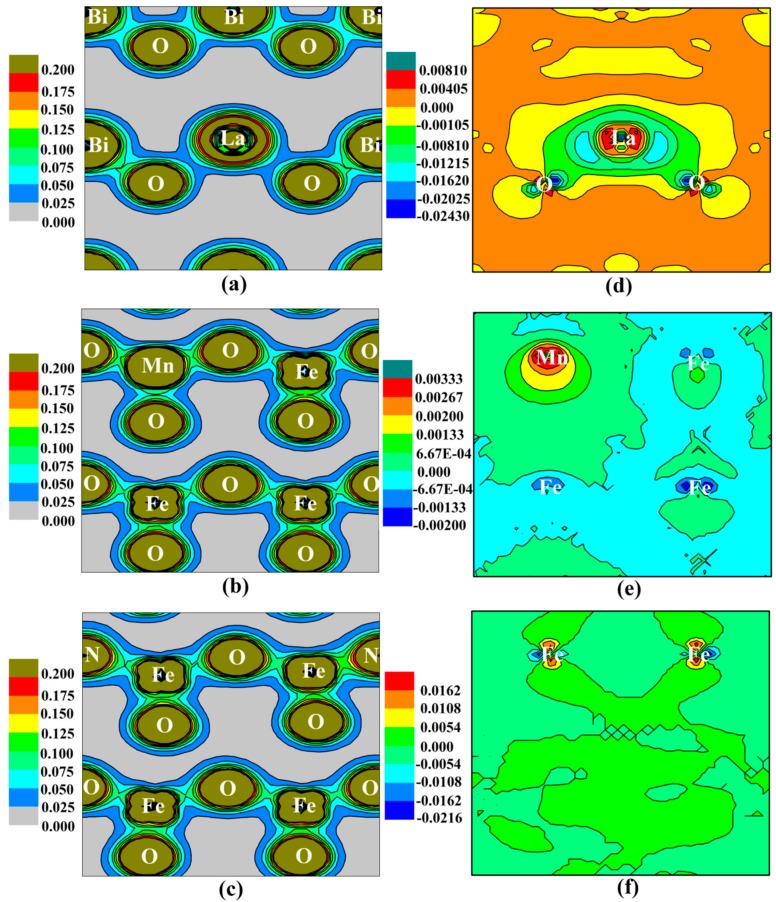
(**a**–**c**) The charge density (e/a.u.^3^) and (**d**–**f**) the difference charge density (e/a.u.^3^) for La-, Mn-, and N-doped tetragonal BFO supercell, respectively.

We next used G-AFM BFO as an example to investigate the origin of spontaneous polarization for La-, Mn-, and N-doped BFO from charge density distribution. Figure 5a–c show the charge density distribution of La-, Mn-, and N-doped BFO, respectively. The ionic interaction between La and O is stronger than that of Bi and O (Figure 5a), the Mn-O and Fe-N covalent interaction is stronger than that of Fe-O at the equivalent position (Figure 5b,c). The difference charge density analysis further demonstrated the charge transfer during the bonding process for La-, Mn-, and N-doped BFO (Figure 5d–f, respectively). Here the difference charge density is got by the difference charge density of La, Mn and N-doped BFO minus that of pure BFO, respectively. From which we can obtain the charge-transfer distribution when La, Mn and N substituted at A-, B- and O-site of BFO, respectively. For La- and Mn-doped BFO, the distribution in charge transfer was asymmetric along the [001] direction, which was the cause of a small change in spontaneous polarization. For N-doped BFO, however, the distribution in charge transfer was symmetrical and therefore showed no change in spontaneous polarization. Therefore, the charge transfer is the main cause of polarization change for La-, Mn-, and N-doped tetragonal BFO.

## 4. Conclusions

In conclusion, we systematically investigated the magnetic properties and spontaneous polarization for La-, Mn-, and N-doped tetragonal BFO using first-principles. In terms of magnetic properties, Mn- and N-doped tetragonal BFO showed enhanced ferromagnetism that was mediated by *p*-*d* hybridization. However, La-doped tetragonal BFO was nonmagnetic. In terms of spontaneous polarization, La and N substitution had little influence on the spontaneous polarization, whereas Mn substitution had a tendency to reduce the spontaneous polarization. However, the spontaneous polarization values remained substantial compared with prototypical ferroelectric materials (BaTiO_3_ and KNbO_3_). Consequently, the enhanced magnetic properties and the large spontaneous polarization values for Mn- and N-doped tetragonal BFO propose a new route for improving the multiferroic properties of BFO film.

## Figures and Tables

**Table 1:** Values of N∙∙∙N distance (*d*_N―N_) (Å), magnetic ordering of ground state, relative energy, and total magnetic moment (*M*_tot_) calculated for each configuration of two nitrogen (N) atoms doped in BiFeO_3_ (BFO) with G-antiferromagnetic (AFM) ordering. The energy difference between ground state of each configuration and C_S3_ configuration is the relative energy. The negative value of magnetic moment denotes its direction is downward.

Configurations	d_N―N_ (Å)	Ground State	Relative Energy (meV)	M_tot_ (μ_B_)
(f, 1)	3.769	AFM	706.5	0
(f, 2)	5.330	FM	674.5	2
(f, 3)	4.946	AFM	733.9	0
(f, 4)	6.218	FM	705.4	2
(f, 5)	7.271	AFM	704.5	0
(s, 1)	2.427	-	43.5	0
(s, 2)	3.769	-	186.8	0
(s, 3)	2.948	FM	0	2
(3, 4)	2.952	FM	354.8	1.99
(s, 4)	4.946	FM	201.5	−1.98
(s, 5)	5.603	FM	91.9	1.97
(s, 6)	6.192	AFM	159.9	0

**Table 2:** The calculated results of each configuration for two N atoms doped in a 2×2×2 tetragonal BFO supercell with C-AFM magnetic lattice.

Configurations	d_N―N_ (Å)	Ground State	Relative Energy (meV)	M_tot_ (μ_B_)
(f, 1)	3.769	AFM	586.7	0
(f, 2)	5.330	FM	586.7	−2
(f, 3)	4.946	FM	778.4	−2
(f, 4)	6.218	AFM	766.7	0
(f, 5)	7.271	FM	690.1	−2
(s, 1)	2.410	-	29.9	0
(s, 2)	3.769	-	211.1	0
(s, 3)	2.923	FM	0	−2
(3, 4)	3.155	FM	348.4	−1.94
(s, 4)	4.946	AFM	249.9	0
(s, 5)	5.639	AFM	119.3	0
(s, 6)	6.204	FM	220.3	1.99

**Table 3:** The calculated results of one La-, Mn and N-doped 2×2×2 tetragonal BFO supercell with G-AFM and C-AFM magnetic lattices.

Compound	Spontaneous Polarization P (µC/cm^2^)		Energy Barrier Height (eV/f.u.)	
	G	C	G	C
12.5% La-doped BFO	154.4	150.3	2.125	2.160
12.5% Mn-doped BFO	145.0	123.8	2.086	2.080
4.17% N-doped BFO	147.3	149.5	2.146	2.176
